# Comparative analysis of the association between traditional and lipid-related obesity indicators and isolated systolic hypertension: Association of obesity indicators with ISH

**DOI:** 10.1186/s12872-022-02564-2

**Published:** 2022-03-21

**Authors:** Jian Dong Qian, Xiao Mei Li, Dong Shui Chen, Jian Qin Zhu, Xing Zhen Liu

**Affiliations:** 1 Department of Cardiology, Jingjiang People’s Hospital, Jiangsu, China; 2 Department of Rheumatology, Special Medical Center of China Air Force, Beijing, China; 3 Hangzhou Aeronautical Sanatorium for Special Service of China Air Force, No. 27, Yang Gong Di, Xihu District, Hangzhou, 310007 Zhejiang China; 4 Department of Rheumatology, Jingjiang People’s Hospital, No. 28, Zhongzhou Road, Jingjiang, 214500 Jiangsu China

**Keywords:** Hypertension, Systolic hypertension, Anthropometric index, Obesity

## Abstract

**Background:**

Obesity is a well-known modified risk factor for isolated systolic hypertension (ISH), but evidence is lacking regarding whether the combination of anthropometric and lipid indicators could strengthen their correlation with ISH. Therefore, we compared the association of body mass index (BMI), waist circumference (WC), waist-to-hip ratio (WHR), waist-to-height ratio (WHtR), visceral adiposity index (VAI), lipid accumulation product index (LAP), and cardiometabolic index (CMI) with ISH.

**Methods:**

A total of 106,248 adults who received routine health screening and did not have diastolic blood pressure ≥ 90 mmHg were recruited in this cross-sectional study. The associations between these indicators and ISH were evaluated using multivariate regression.

**Results:**

Each standard deviation (SD) increase in traditional obesity indicators (especially WHR and WHtR) had significantly higher multivariate-adjusted odds ratios (ORs) than each SD increase in lipid-related obesity indicators. In addition, multivariate-adjusted ORs for ISH in the third (vs. the first) tertile of traditional obesity indicators were also significantly higher than those of lipid-related indicators. Moreover, traditional obesity indicators exhibited a higher area under the ROC curve for discriminating ISH than lipid-related obesity indicators.

**Conclusions:**

Traditional obesity indicators were more strongly associated with ISH than lipid-related obesity indicators among Chinese adults.

**Supplementary Information:**

The online version contains supplementary material available at 10.1186/s12872-022-02564-2.

## Introduction

Isolated systolic hypertension (ISH) is defined as systolic blood pressure (SBP) ≥ 140 mmHg and diastolic blood pressure (DBP) < 90 mmHg. ISH is the most common hypertension phenotype in elderly individuals and is also often present in young and middle-aged individuals [1]. Numerous studies have demonstrated that ISH has an important role in predicting cardiovascular risk and all-cause mortality in both young and elderly adults [2, 3]. Obesity is also a well-established and modifiable risk factor for hypertension [4]. Insulin resistance (IR) and a chronic inflammatory state caused by obesity may contribute to ISH [5]. Accordingly, weight monitoring and active intervention are critical for the prevention and management of ISH.

Anthropometry is the most cost-effective tool for assessing obesity. Traditional obesity indicators, including body mass index (BMI), waist circumference (WC), waist-to-hip ratio (WHR), and waist-to-height ratio (WHtR), are the most widely accepted and valuable anthropometric measures for adiposity. In addition to these traditional indicators, some novel obesity indicators based on anthropometric and lipid parameters [visceral adiposity index (VAI), lipid accumulation product index (LAP), and cardiometabolic index (CMI)] have also been proposed.

Although several studies have demonstrated that these novel lipid-related obesity indicators were positively associated with hypertension [6], there are no data on their association with ISH and whether they have an advantage over traditional indicators. Thus, we conducted this large-scale cross-sectional study to compare the association of traditional and lipid-related obesity indicators with ISH. This study aims to provide more clinical evidence for the selection of obesity indicators when monitoring and managing the weight of patients with ISH.

## Methods

### Study population

This cross-sectional study was based on a database of adults who received routine health screening between January 2013 and July 2019 in China, which has been previously described [7, 8]. The main exclusion criteria were DBP ≥ 90 mmHg and the use of statins and antihypertensive drugs. Ultimately, 106,248 adults were eligible for this analysis.

### Basic data collection

There was a process of collecting basic information before the health screening that included family history, medical history, basic diseases, medications, smoking and alcohol consumption. SBP, DBP, and heart rate (HR) were obtained 3 times on the right arm after at least 5 min of rest using an automatic BP monitor (HEM-1000, OMRON, Japan). ISH was defined as having an SBP ≥ 140 mmHg and DBP < 90 mmHg, and nonhypertension was defined as having a blood pressure (BP) less than 140/90 mmHg. Considering that age plays an important role in ISH, we grouped the individuals by age for the analysis, and the cutoff point for age grouping was 50 years old.

### Biochemical tests

Blood samples were collected after a minimum of 8 h of overnight fasting. Serum levels of fasting plasma glucose (FPG), plasma uric acid (UA), total cholesterol (TC), triglyceride (TG), low-density lipoprotein cholesterol (LDLc), and high-density lipoprotein cholesterol (HDLc) were measured by a biochemical autoanalyzer.

### Anthropometric measurement

Height and weight were measured for subjects who were barefoot and in light clothing on electronic scales. WC and hip circumference (HC) were measured according to a standardized protocol and technique by well-trained nurses. BMI was calculated as weight divided by the square of height. The WHR was calculated as WC divided by the HC; the WHtR was calculated as WC divided by height. The lipid-related obesity indicator calculation formulas were as follows: ${\text{VAI}}\;{\text{(females)}} = \left( {\frac{WC}{{36.58 + 1.89 \times BMI}}} \right) \times \left( {\frac{TG}{{0.81}}} \right) \times \left( {\frac{1.52}{{HDLc}}} \right)$, ${\text{VAI}}\;{\text{(males)}} = \left( {\frac{WC}{{39.68 + 1.88 \times BMI}}} \right) \times \left( {\frac{TG}{{1.03}}} \right) \times \left( {\frac{1.31}{{HDLc}}} \right)$ [9]; ${\text{LAP}}\;{\text{(females)}} = (WC - 58) \times TG$, ${\text{LAP}}\;{\text{(males)}} = (WC - 65) \times TG$ [10]; and ${\text{CMI}} = \frac{TG}{{HDLc}} \times {\text{WHtR}}$ [11].

### Statistical analysis

The data are expressed as numbers or means ± SD. Categorical variables, such as the frequency of smoking and drinking, were compared with the chi-square test. The t test was used to compare continuous variables, such as obesity indicators and biochemical parameters, between the two groups. A correlation matrix chart was used to show the distribution of data and the correlation between BP and obesity indicators and lipid parameters (TG and HDLc).

Logistic regression analyses were applied to explore the associations between obesity indicators and ISH after adjusting for the confounding effects of age, sex, hyperuricemia, diabetes, alcohol intake, and smoking status. The performance of obesity indicators both as continuous variables and categorical variables (dummy variable) in the regression equation was examined. When target indicators were used as a categorical variable, traditional and lipid-related obesity indicators and lipid parameters (TG and HDLc) were divided into tertiles, and the lowest tertile was used as a reference in the regression analysis. In addition, a logistic regression with cubic spline functions and smooth curve fitting (restricted cubic spline) were used to examine the nonlinear association between obesity indicators and ISH. Before that, a natural log-transformed was performed to normalize the distribution of TG, HDLc, and three lipid-related obesity indicator.

Receiver operating characteristic (ROC) analyses and the area under the ROC curve (AUC) were used to evaluate the ability of these indicators to distinguish ISH. In general, an AUC between 0.5 and 0.6 represents bad accuracy but remains useful for discrimination, and with AUC from 0.6 to 0.7, 0.7 to 0.8, 0.8 to 0.9, and 0.9–1 suggests sufficient, good, very good, and excellent discrimination ability, respectively [12]. Statistical analyses were performed using SPSS version 18.0 (SPSS Inc., Chicago, Illinois, USA) or R 4.0.5 (R Foundation for Statistical Computing, Vienna, Austria). A *P* value < 0.05 was considered statistically significant.

## Results

Of the 106,248 adults with DBP < 90 mmHg, 40.3% were women, 65.4% were younger adults (less than 50 years old), and the mean age was 45.6 ± 13.1 years. The basic clinical characteristics of the included individuals are shown in Table 1. The proportion of ISH was 4.4% among younger adults and 20.3% among older adults. In both age groups, patients with ISH were older and had higher HR, FPG, UA, TC, TG, and obesity indicators (except WC in younger adults) than nonhypertensive patients.

**Table 1:** The basic clinical characteristics of study subjects according to age

Characteristics	Younger adults			Older adults		
	Non-ISH	ISH	*p* value	Non-ISH	ISH	*p* value
No., n (%)	66,398	3066 (4.4%)	< 0.001	29,314	7470 (20.3%)	< 0.001
Women (%)	41.7%	19.8%	< 0.001	39.2%	40.2%	0.128
Age (y)	37.9 ± 7.4	38.3 ± 7.9	0.001	58.8 ± 6.9	65.7 ± 9.5	< 0.001
Smoking (%)	14.0%	16.7%	0.093	21.6%	14.7%	0.067
Drinking (%)	12.1%	16.8%	0.036	17.9%	16.3%	0.172
SBP (mmHg)	116.0 ± 12.0	145.5 ± 5.8	< 0.001	121.0 ± 11.4	150.7 ± 10.1	< 0.001
DBP (mm Hg)	71.6 ± 8.9	82.6 ± 5.5	< 0.001	74.3 ± 8.6	80.5 ± 6.9	< 0.001
HR (beats/min)	79.9 ± 12.1	86.1 ± 14.2	< 0.001	76.8 ± 11.3	79.9 ± 13.3	< 0.001
FPG (mmol/L)	5.46 ± 0.78	5.76 ± 1.11	< 0.001	6.00 ± 1.35	6.52 ± 1.73	< 0.001
Type 2 diabetes (%)	2.0%	5.6%	< 0.001	10.8%	21.4%	< 0.001
UA (μmol/L)	336.3 ± 88.6	375.0 ± 88.3	0.017	343.6 ± 83.5	350.9 ± 84.4	< 0.001
Hyperuricemia (%)	19.3%	30.7%	< 0.001	17.6%	20%	< 0.001
TC (mmol/L)	4.64 ± 0.85	4.84 ± 0.90	< 0.001	4.98 ± 0.94	5.02 ± 0.98	< 0.001
TG (mmol/L)	1.38 ± 1.13	1.77 ± 1.45	< 0.001	1.62 ± 1.21	1.72 ± 1.32	< 0.001
HDLc (mmol/L)	1.51 ± 0.34	1.41 ± 0.31	< 0.001	1.49 ± 0.36	1.50 ± 0.35	0.786
LDLc (mmol/L)	2.48 ± 0.70	2.64 ± 0.72	0.146	2.71 ± 0.77	2.69 ± 0.79	0.198
BMI (kg/m^2^)	23.1 ± 3.2	25.4 ± 3.6	< 0.001	24.0 ± 2.9	24.9 ± 3.1	< 0.001
WC (cm)	77.5 ± 9.6	84.2 ± 10.0	< 0.001	81.6 ± 8.9	84.2 ± 9.3	< 0.001
WHR	0.83 ± 0.07	0.87 ± 0.07	< 0.001	0.87 ± 0.07	0.89 ± 0.07	< 0.001
WHtR	0.46 ± 0.05	0.50 ± 0.05	< 0.001	0.50 ± 0.05	0.52 ± 0.05	< 0.001
VAI	1.43 ± 0.66	1.89 ± 0.45	< 0.001	1.77 ± 0.87	1.92 ± 0.27	< 0.001
LAP	24.7 ± 10.5	40.5 ± 15.2	< 0.001	33.9 ± 12.8	40.1 ± 18.7	< 0.001
CMI	0.51 ± 0.36	0.73 ± 0.32	< 0.001	0.63 ± 0.31	0.68 ± 0.29	< 0.001

ISH, isolated systolic hypertension; SBP, systolic blood pressure; DBP, diastolic blood pressure; HR, heart rate; FPG, fasting plasma glucose; UA, plasma uric acid; TC, total cholesterol; TG, triglyceride; HDLc, high-density lipoprotein cholesterol; LDLc, low-density lipoprotein cholesterol; BMI, body mass index; WC, waist circumference; WHR, waist to hip ratio; WHtR, waist to height ratio; VAI, visceral adiposity index; LAP, lipid accumulation product index; CMI, cardiometabolic index

As shown in the correlation matrix chart (Additional file 1: Fig. S1), the correlation coefficients between traditional indicators and BP values were greater than those of lipid-related obesity indicators and lipid parameters. The proportion of ISH also showed a significant increasing trend with the rise of obesity indicators and lipid parameters (Additional file 1: Fig. S2). This trend of increasing ISH proportions was more pronounced in younger adults.

**Fig. 1:**
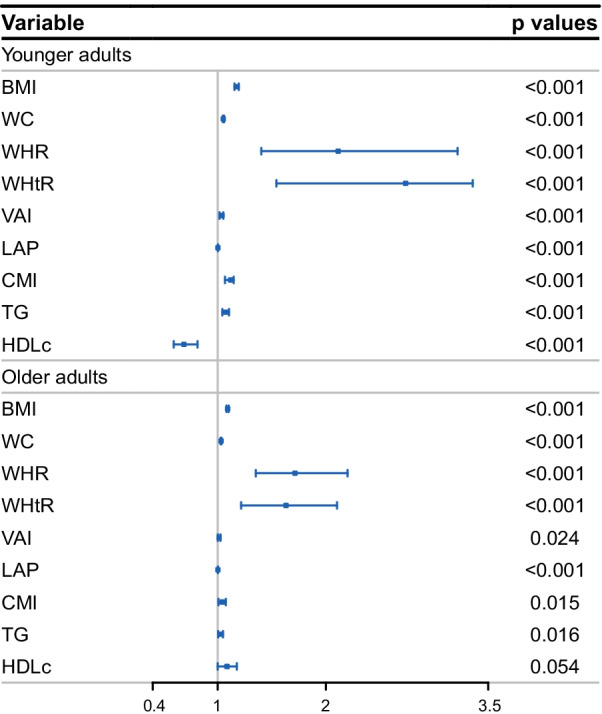
Multivariate-adjusted odds ratios for ISH by each standard deviation increase of indicators. ISH, isolated systolic hypertension; BMI, body mass index; WC, waist circumference; WHR, waist-to-hip ratio; WHtR, waist-to-height ratio; VAI, visceral adiposity index; LAP, lipid accumulation product index; CMI, cardiometabolic index; TG, triglyceride; HDLc, high-density lipoprotein cholesterol; adjusted covariables included age, sex, hyperuricemia, diabetes, alcohol intake, and smoking status

**Fig. 2:**
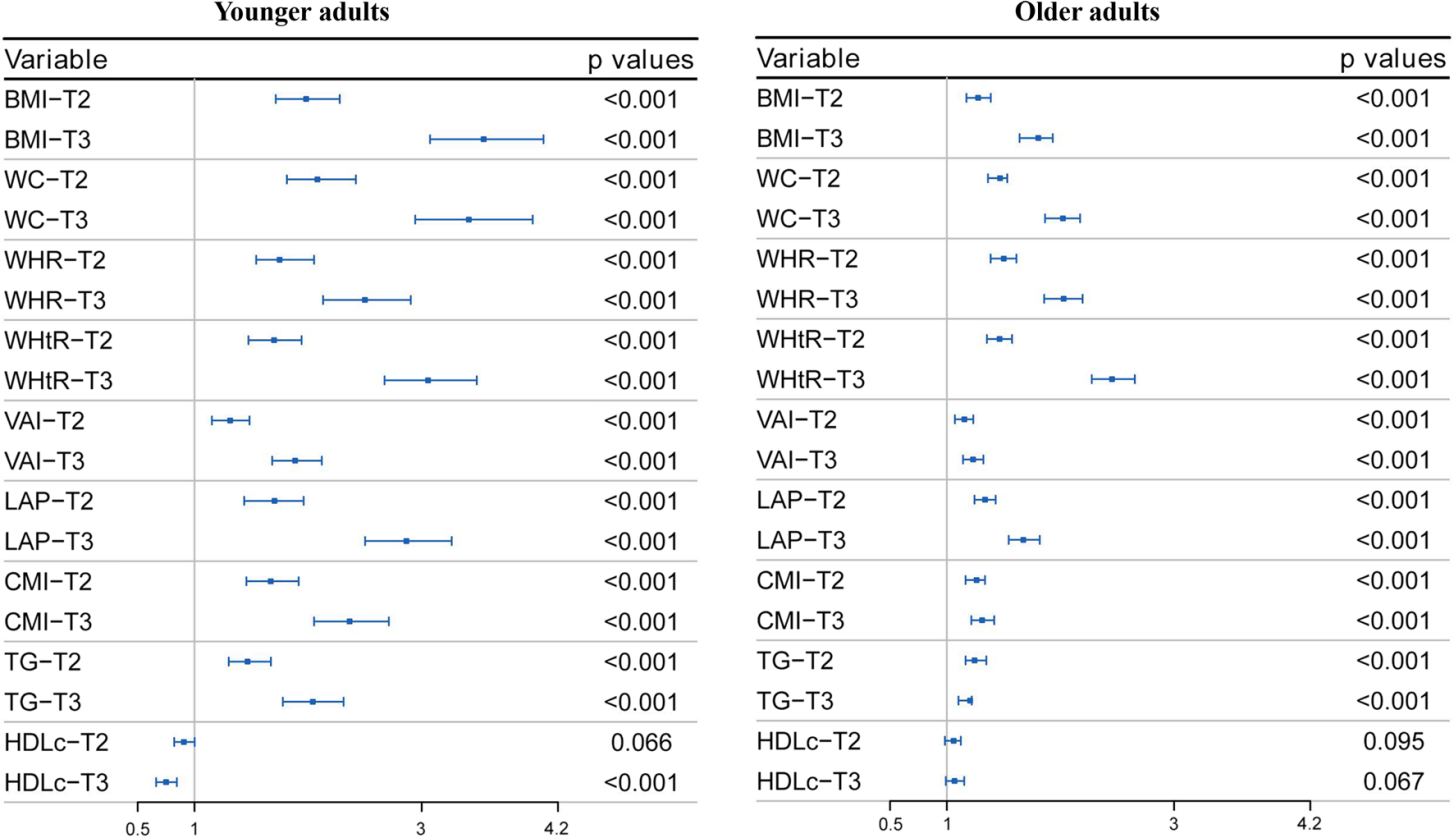
Multivariate-adjusted odds ratios for ISH in the second and third (vs. the first) tertiles of each indicator. ISH, isolated systolic hypertension; BMI, body mass index; WC, waist circumference; WHR, waist-to-hip ratio; WHtR, waist-to-height ratio; VAI, visceral adiposity index; LAP, lipid accumulation product index; CMI, cardiometabolic index; TG, triglyceride; HDLc, high-density lipoprotein cholesterol; T2, second tertile; T3, the highest tertile; adjusted covariables included age, sex, hyperuricemia, diabetes, alcohol intake, and smoking status

**Fig. 3:**
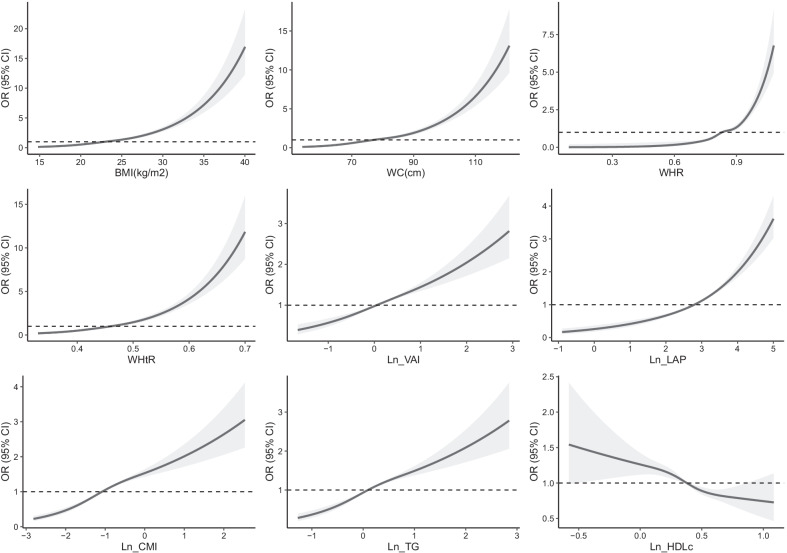
Association between insulin resistance indicators and ISH using cubic smoothing splines in younger adults. Odds ratios, OR; ISH, isolated systolic hypertension; BMI, body mass index; WC, waist circumference; WHR, waist-to-hip ratio; WHtR, waist-to-height ratio; VAI, visceral adiposity index; LAP, lipid accumulation product index; CMI, cardiometabolic index; TG, triglyceride; HDLc, high-density lipoprotein cholesterol; adjusted covariables included age, sex, hyperuricemia, diabetes, alcohol intake, and smoking status

**Fig. 4:**
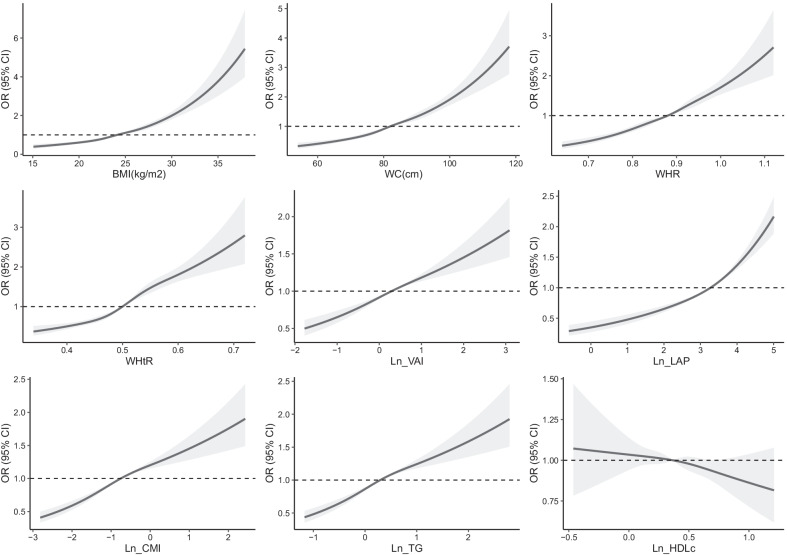
Association between insulin resistance indicators and ISH using cubic smoothing splines in older adults. Odds ratios, OR; ISH, isolated systolic hypertension; BMI, body mass index; WC, waist circumference; WHR, waist-to-hip ratio; WHtR, waist-to-height ratio; VAI, visceral adiposity index; LAP, lipid accumulation product index; CMI, cardiometabolic index; TG, triglyceride; HDLc, high-density lipoprotein cholesterol; adjusted covariables included age, sex, hyperuricemia, diabetes, alcohol intake, and smoking status

The logistic regression analysis results are shown in Figs. 1 and 2. The results showed that each SD increase in obesity indicators and TGs had significant multivariate-adjusted odds ratios (ORs) for ISH in both younger and older adults. Each SD increase in WHR and WHtR had a significantly higher OR than each SD increase in lipid-related obesity indicators (Fig. 1). Then, the subjects were classified into tertiles according to each obesity indicator and lipid parameter. Multivariate-adjusted ORs for ISH in the second and third (vs. the first) tertiles of each indicator are shown in Fig. 2. BMI had the highest adjusted OR for ISH among younger adults, and the OR in the third (vs. first) tertile was 3.546 (95% CI 3.074–4.072). WHtR had the highest adjusted OR among older adults, and the OR in the third (vs. first) tertile was 2.454 (95% CI 2.276–2.655). All other corresponding OR values are displayed in Additional file 1: Tables S1 and S2. The association between the three IR indicators and the ISH using cubic smoothing splines are shown in Figs. 3 and 4.

Although lipid-related obesity indicators (especially LAP) exhibited a higher AUC for discriminating ISH than their composition (TG and HDLc) (Additional file 1: Fig. S3), the AUC values for lipid-related obesity indicators were significantly lower than traditional indicators (Additional file 1: Fig. S4). BMI had the largest AUC for ISH [0.689 (95% CI 0.679–0.698)] in younger adults, and WHtR had the largest AUC for ISH [0.621 (95% CI 0.614–0.628)] in older adults. The corresponding AUC values for obesity indicators, TG, and HDLc as they relate to ISH are presented in Additional file 1: Table S3.

## Discussion

The present study compared the strength of the associations of traditional and lipid-related obesity indicators with ISH and found that the association of traditional obesity indicators with ISH was more prominent than that of lipid-related obesity indicators. BMI and WHtR showed the best performance in younger and older adults, respectively.

Obesity is a well-known determinant for ISH [13]. The association of body weight and BP was recognized at the beginning of the twentieth century and demonstrated prospectively in the Framingham Heart Study [14]. It was not until the 1980s that there was a clear explanation of the correlation between weight and BP. The potential mechanisms involved in the association between obesity and the development of ISH include IR, low-grade inflammation, endothelial dysfunction, excess salt intake, and increased salt sensitivity, which may contribute to increased arterial stiffness and cardiac output (CO) [5].

Although the mechanism by which obesity participates in ISH has been gradually recognized, the heterogeneity of obesity and the diversity of assessment tools make it unclear which obesity indicators have the strongest association with ISH. Obesity indicators included in this study also showed differences in their associations with ISH. Furthermore, their associations with ISH varied according to age, which is not surprising because ISH itself is an age-related disease and has different pathophysiological mechanisms at different ages. Age-related changes in peripheral SBP are characterized by a steep increase before the age of 20 years, a plateau phase between 20 and 50 years, and a slow linear climb after 50 years of age [15]. Unlike ISH in elderly individuals, which is related to increased arterial stiffness and reduced aortic diameter, ISH in younger adults may be mainly attributed to increased CO [16].

Given that BMI is the most commonly used indicator of obesity, the association between BMI and ISH has been widely studied. Most studies have observed that a high BMI, age and smoking are determinants of ISH [17]. In the present study, BMI had the highest adjusted OR and AUC values only in younger adults. There are several possible explanations for this result. As mentioned earlier, increased CO may be the main pathophysiological mechanism of ISH in younger adults. Due to the increased tissue mass, an increase in CO is needed to sustain metabolic requirements [18], so CO is closely associated with body size. In fact, the increased CO in obese individuals is mainly due to the increase in metabolically active fat-free mass (FFM) [19]. The weak ability of BMI to distinguish between FFM and adipose tissue makes BMI more strongly correlated with CO than other obesity indicators [20], which contributes to the outstanding association of BMI with ISH in younger adults.

Another finding of this study was that WHR and WHtR were highly associated with ISH in both age categories. Although arterial stiffness occurs mostly in the elderly, the associations between abdominal obesity and arterial stiffness may already be present by age 36 or earlier [21], which could partially explain the better performance of WHR and WHtR in all age categories in this study. In addition to arterial stiffness, abdominal obesity is also associated with CO. de Simone et al. observed that the increased CO in overweight and nonobese individuals was not fully explained by FFM but was also attributable to the abdominal fat distribution [22]. Furthermore, candidate mechanisms by which abdominal fat contributes to ISH also include IR and low-grade inflammation [23].

Unexpectedly, despite the combination of lipid parameters, the performance of lipid-related obesity indicators were mediocre in this study. Theoretically, the obesity index combined with lipid parameters could better reflect visceral obesity because of the close relationship between lipid metabolism and visceral fat [9–11]. Because excess visceral fat is more likely to cause IR and chronic inflammation than subcutaneous fat [24], more robust associations between lipid-related obesity indicators and ISH should also be observed [25]. Nonetheless, these indicators did not show a better association with ISH than traditional ones.

In line with our findings, several previous studies demonstrated superiority of traditional obesity indicators than lipid-related ones in other cardiometabolic risk factors. Kavaric and colleagues found that VAI and LAP were not better than WC for type 2 diabetes mellitus prediction [26]. Another comparative study based on African populations also showed that VAI and LAP were not better than WC for predicting Metabolic Syndrome in both men and women [27].

Considering that TG and/or HDLc are used in the calculation formula of lipid-related indicators, so we also examined the association of TG and HDLc with ISH. In the elderly group, the association between HDLc and ISH was not statistically significant. In the younger group, HDLc was generally negatively associated with ISH. However, it can be intuitively seen from the fitted curve that HDLc was negatively associated with ISH only when HDLc was at a moderate level (Fig. 3), and the significance of the association of HDLc with ISH disappeared with further increased in HDLc levels. Therefore, the insignificant association of HDLc with ISH in the elderly group and its unstable association with ISH in the younger group may partially contribute to the moderate association between lipid-related indicators and ISH. (reviewer #1, comment #2).

We attempted to speculate on possible explanations for these results in terms of visceral fat, since lipid-related indicators were designed to predict visceral adiposity. A recent study showed that WC and BMI were more strongly associated with MRI-derived fat compartments than HDLc [28], which indicates that the combination of lipid parameters may not increase the association of traditional anthropometric parameters with visceral adiposity. Borruel et al. [29] reported that WC and BMI showed more correlations with ultrasound measurements of visceral adiposity than that of VAI. These properties of lipid parameters themselves and lipid-related indicators may have contributed to their performance in this study.

Several potential limitations of this study should be mentioned. First, the subjects of this study were from China, which makes it difficult to generalize our results to other ethnic populations. Second, the cross-sectional study design was unable to show the causal associations between these obesity indicators and ISH. Future longitudinal research is needed to explore which obesity index can be used as a predictor of ISH. Third, according to ROC analyses, the ability of the included obesity indicators to distinguish ISH was relatively weak (all AUC < 0.7). In fact, directly measuring BP is more accurate and convenient than using other indirect indicators to distinguish ISH. The purpose of ROC analysis in this study was to further compare the association of obesity indicators with ISH instead of evaluating their ability to discriminate ISH. Therefore, the ROC analysis results must be interpreted with caution.

## Conclusions

Compared with lipid-related obesity indicators, traditional ones such as BMI, WC, WHR, and WHtR were more associated with ISH among Chinese adults. Therefore, these simple and economical anthropometric indicators can still play a role in the weight monitoring among ISH individuals.

## Supplementary Information

**Additional file 1**. Supplementary figures and tables.

## Data Availability

The datasets used and/or analyzed during the current study are available from the corresponding author on reasonable request.
