# Supplementary material for: Comparative analysis of the association between traditional and lipid-related obesity indicators and isolated systolic hypertension: Association of obesity indicators with ISH
Source: BMC Cardiovasc Disord. 2022 Mar 21;22:119. doi: 10.1186/s12872-022-02564-2 (PMC8939084; doi:10.1186/s12872-022-02564-2)
Supplement: Supplementary file 1 — Additional file 1. Supplementary figures and tables. [file 12872_2022_2564_MOESM1_ESM.pdf]

S Table 1 Multivariate-adjusted odds ratios and 95% confidence interval for ISH by each standard deviation increase of indicators.

Variable	Younger adults				Older adults			
	OR	LL	UL	*P* values	OR	LL	UL	*P* values
**BMI**	1.178	1.156	1.196	<0.001	1.093	1.082	1.102	<0.001
**WC**	1.052	1.049	1.061	<0.001	1.032	1.029	1.036	<0.001
**WHR**	2.114	1.404	3.217	<0.001	1.715	1.353	2.201	<0.001
**WHtR**	2.737	1.543	3.357	<0.001	1.632	1.217	2.103	<0.001
**VAI**	1.041	1.017	1.053	<0.001	1.014	1.002	1.027	0.024
**LAP**	1.003	1.002	1.005	<0.001	1.003	1.002	1.004	<0.001
**CMI**	1.120	1.069	1.148	<0.001	1.044	1.009	1.076	0.015
**TG**	1.076	1.044	1.105	<0.001	1.025	1.005	1.05	0.016
**HDLc**	0.689	0.594	0.815	<0.001	1.087	1.000	1.177	0.054

ISH, isolated systolic hypertension; BMI, body mass index; WC, waist circumference; WHR, waist to hip ratio; WHtR, waist to height ratio; VAI, visceral adiposity index; LAP, lipid accumulation product index; CMI, cardiometabolic index; TG, triglyceride; HDLc, high-density lipoprotein cholesterol; LL and UL, lower level and upper level of 95% confidence interval.

S Table 2 Multivariate-adjusted odds ratios and 95% confidence interval for ISH in the second and third (vs the first) tertile of each indicator.

Variable	Younger adults				Older adults			
	**OR**	**LL**	**UL**	***P* values**	**OR**	**LL**	**UL**	***P* values**
BMI-T2	1.983	1.715	2.279	<0.001	1.275	1.173	1.387	<0.001
BMI-T3	3.546	3.074	4.072	<0.001	1.805	1.640	1.932	<0.001
WC-T2	2.082	1.813	2.421	<0.001	1.468	1.362	1.531	<0.001
WC-T3	3.415	2.943	3.977	<0.001	2.022	1.864	2.173	<0.001
WHR-T2	1.749	1.543	2.053	<0.001	1.501	1.385	1.612	<0.001
WHR-T3	2.500	2.132	2.904	<0.001	2.028	1.857	2.195	<0.001
WHtR-T2	1.701	1.475	1.943	<0.001	1.464	1.352	1.574	<0.001
WHtR-T3	3.056	2.673	3.485	<0.001	2.454	2.276	2.655	<0.001
VAI-T2	1.314	1.153	1.485	<0.001	1.154	1.071	1.232	<0.001
VAI-T3	1.886	1.684	2.121	<0.001	1.231	1.142	1.321	<0.001
LAP-T2	1.704	1.437	1.961	<0.001	1.336	1.243	1.429	<0.001
LAP-T3	2.866	2.501	3.264	<0.001	1.673	1.544	1.818	<0.001
CMI-T2	1.671	1.457	1.916	<0.001	1.262	1.164	1.335	<0.001
CMI-T3	2.365	2.053	2.711	<0.001	1.310	1.215	1.417	<0.001
TG-T2	1.467	1.302	1.673	<0.001	1.244	1.165	1.347	<0.001
TG-T3	2.042	1.778	2.312	<0.001	1.205	1.102	1.219	<0.001
HDLc-T2	0.906	0.821	1.001	0.066	1.060	0.983	1.123	0.095
HDLc-T3	0.750	0.662	0.844	<0.001	1.068	0.991	1.153	0.067

ISH, isolated systolic hypertension; BMI, body mass index; WC, waist circumference; WHR, waist to hip ratio; WHtR, waist to height ratio; VAI, visceral adiposity index; LAP, lipid accumulation product index; CMI, cardiometabolic index; TG, triglyceride; HDLc, high-density lipoprotein cholesterol; T2, second tertile; T3, the highest tertile; LL and UL, lower level and upper level of 95% confidence interval.

S Table 3 The AUC with its 95% confidence interval (C.I.) for distinguishing ISH by obesity indicators and lipid parameters

Variable	Younger adults			Older adults		
	**AUC**	**95% C.I.**	***P* values**	**AUC**	**95% C.I.**	***P* values**
**BMI**	0.689	0.679-0.698	<0.001	0.582	0.575-0.59	<0.001
**WC**	0.685	0.676-0.695	<0.001	0.580	0.572-0.587	<0.001
**WHR**	0.659	0.65-0.669	<0.001	0.577	0.57-0.585	<0.001
**WHtR**	0.673	0.663-0.682	<0.001	0.621	0.614-0.628	<0.001
**VAI**	0.608	0.598-0.618	<0.001	0.533	0.526-0.54	<0.001
**LAP**	0.666	0.656-0.675	<0.001	0.571	0.564-0.578	<0.001
**CMI**	0.643	0.633-0.652	<0.001	0.542	0.535-0.55	<0.001
**TG**	0.627	0.617-0.637	<0.001	0.534	0.527-0.541	<0.001
**HDLc**	0.589	0.579-0.599	<0.001	0.501	0.494-0.509	0.746

ISH, isolated systolic hypertension; AUC, area under the receiver-operating characteristic curve; C.I., confidence interval; BMI, body mass index; WC, waist circumference; WHR, waist to hip ratio; WHtR, waist to height ratio; VAI, visceral adiposity index; LAP, lipid accumulation product index; CMI, cardiometabolic index.

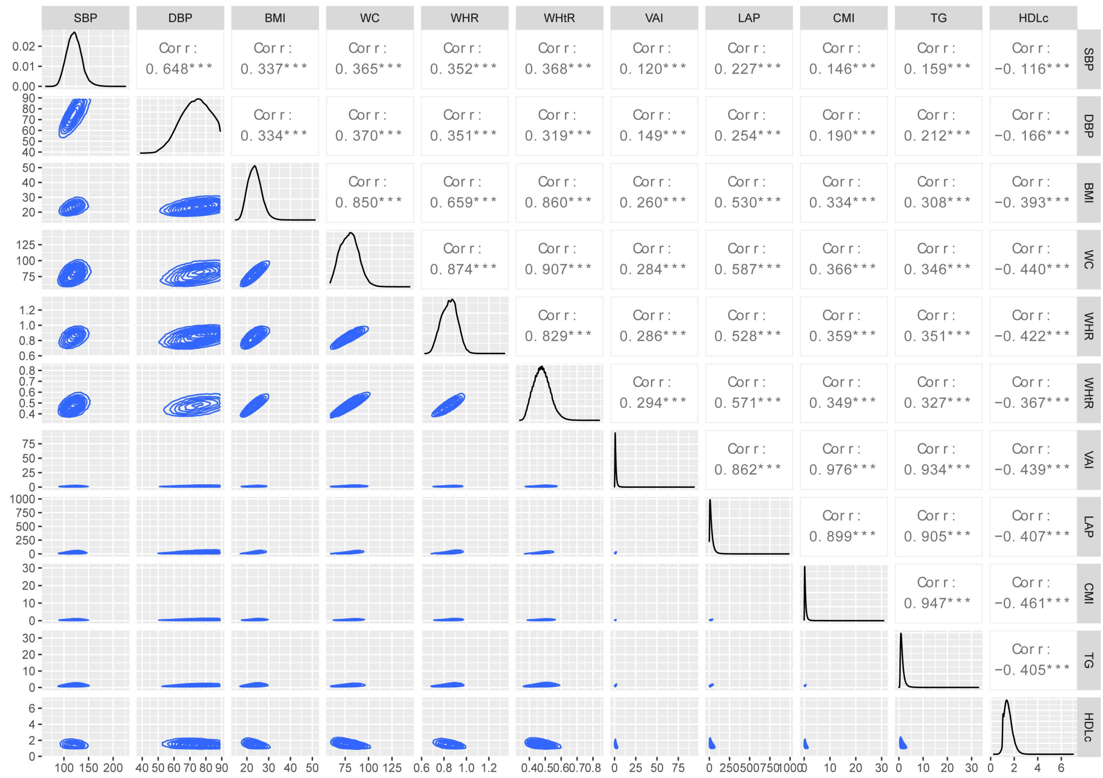

S Figure 1. Correlation matrix diagram between variables.

The lower left corner is the density map between the variables, the diagonal line is the distribution curve of the variables, and in the upper right corner is the correlation coefficient between the variables. SBP, systolic blood pressure; DBP, diastolic blood pressure; TG, triglyceride; HDLc, high-density lipoprotein cholesterol; BMI, body mass index; WC, waist circumference; WHR, waist-to-hip ratio; WHtR, waist-to-height ratio; VAI, visceral adiposity index; LAP, lipid accumulation product index; CMI, cardiometabolic index.

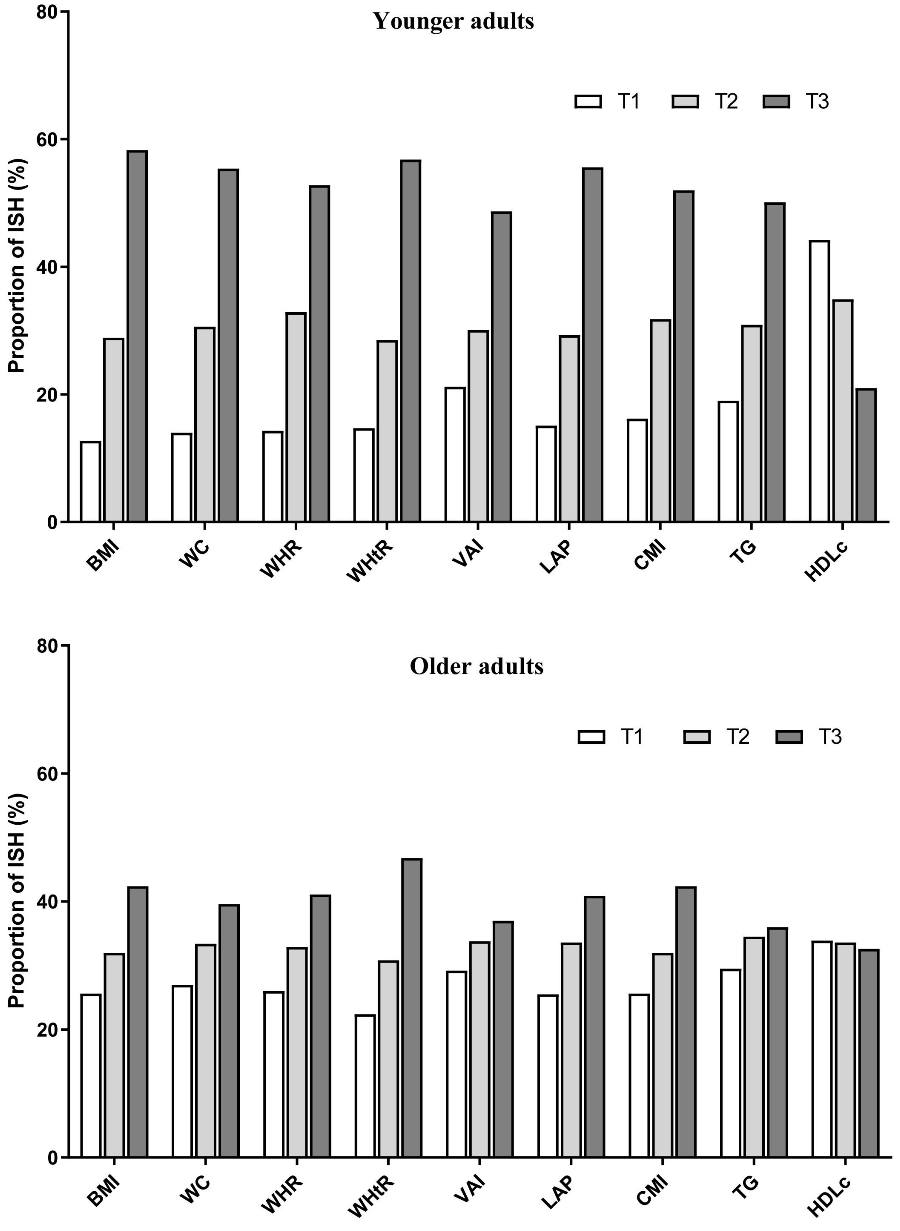

S Figure 2. The proportion of ISH by categories of obesity indicators in different age groups. The subjects were classified into tertiles (T) according to each obesity indicator and lipid parameter: T1, the lowest tertile; T2, second tertile; T3, the highest tertile. ISH, isolated systolic hypertension; BMI, body mass index; WC, waist circumference; WHR, waist-to-hip ratio; WHtR, waist-to-height ratio; VAI, visceral adiposity index; LAP, lipid accumulation product index; CMI, cardiometabolic index; TG, triglyceride; HDLc, high-density lipoprotein cholesterol.

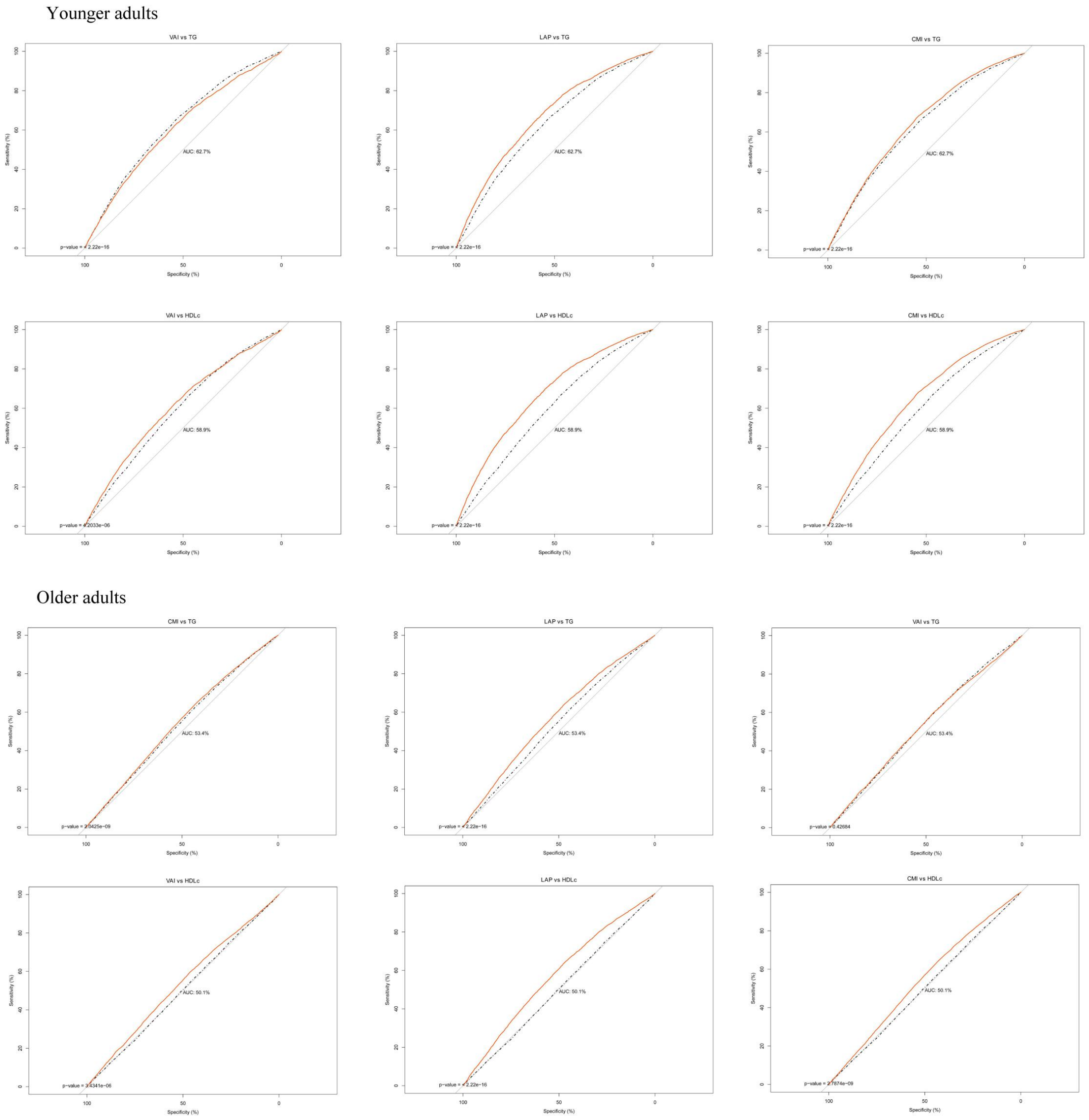

S Figure 3. Receiver operating characteristic (ROC) curves of lipid-related obesity indicators, TG, and HDLc to identify isolated systolic hypertension. The dotted line is the ROC curve of TG or HDLc, and their AUCs are marked next to the diagonal line. The solid red line is the ROC curve of the lipid-related obesity indicator, and in the lower left corner is the *p value* of the comparisons of their AUCs. VAI, visceral adiposity index; LAP, lipid accumulation product index; CMI, cardiometabolic index; TG, triglyceride; HDLc, high-density lipoprotein cholesterol.

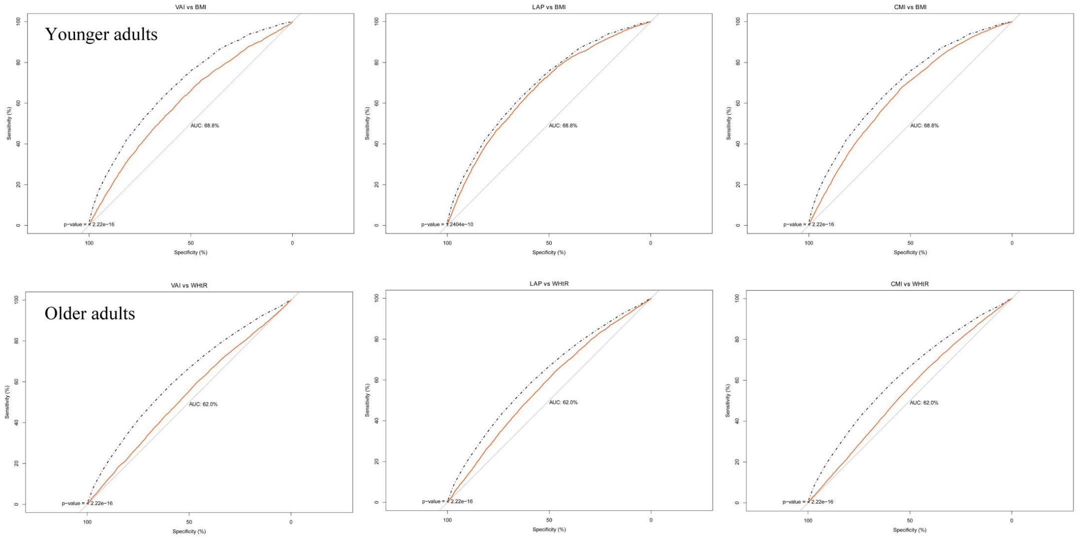

S Figure 4. Receiver operating characteristic (ROC) curves of lipid-related obesity indicators and typical traditional obesity indicators to identify isolated systolic hypertension. The dotted line is the ROC curve of the traditional obesity indicators with the highest AUC in each age group (BMI in younger adults and WHtR in older adults), and their AUCs are marked next to the diagonal line. The solid red line is the ROC curve of the lipid-related obesity indicators, and in the lower left corner is the *p value* of the comparisons of their AUCs. BMI, body mass index; WHtR, waist-to-height ratio; VAI, visceral adiposity index; LAP, lipid accumulation product index; CMI, cardiometabolic index.
